# Age, gender, quadriceps strength and hop test performance are the most important factors affecting the achievement of a patient-acceptable symptom state after ACL reconstruction

**DOI:** 10.1007/s00167-019-05576-2

**Published:** 2019-06-22

**Authors:** Riccardo Cristiani, Christina Mikkelsen, Gunnar Edman, Magnus Forssblad, Björn Engström, Anders Stålman

**Affiliations:** 1 Department of Molecular Medicine and Surgery, Stockholm Sports Trauma Research Center, Karolinska Institutet, Stockholm, Sweden; 2 Capio Artro Clinic, FIFA Medical Centre of Excellence, Sophiahemmet Hospital, Valhallavägen 91, 11486 Stockholm, Sweden

**Keywords:** Anterior cruciate ligament, ACL reconstruction, KOOS, Subjective knee function, Rehabilitation, Quadriceps strength, Single-leg-hop test

## Abstract

**Purpose:**

To assess the percentage of patients achieving an acceptable symptom state 2 years after primary anterior cruciate ligament reconstruction (ACLR) and to identify factors affecting its achievement, in a large cohort.

**Methods:**

Patients who underwent primary ACLR at Capio Artro Clinic, Stockholm, Sweden, from 2005 to 2015, were identified in our clinic registry. Patients who had completed the Knee injury and Osteoarthritis Outcome Score (KOOS) at the 2-year follow-up were included. The primary outcome was the achievement of a patient-acceptable symptom state (PASS) for each KOOS subscale. A multivariate logistic regression analysis was used to determine whether patient age, gender, time from injury to surgery, pre-injury Tegner activity level, graft type, cartilage injury, the presence of medial meniscus (MM) or lateral meniscus (LM) resection or repair and the recovery of 6-month symmetrical (limb symmetry index [LSI] of ≥ 90%) isokinetic quadriceps or hamstring strength and single-leg-hop test performance were factors associated with the achievement of a PASS for each KOOS subscale.

**Results:**

A total of 2335 primary ACLRs were included. More than 60% of the patients reported a PASS on four of the five KOOS subscales. Age ≥ 30 years and an LSI of ≥ 90% for 6-month isokinetic quadriceps strength increased the odds of achieving a PASS across all KOOS subscales. Female gender reduced the odds of achieving a PASS on the Pain (OR 0.76; 95% CI 0.62–0.94; *P* = 0.01), activities of daily living (ADL) (OR 0.79; 95% CI 0.64–0.97; *P* = 0.02) and sport and recreation (OR 0.72; 95% CI 0.58–0.89; *P* = 0.003) subscales. The presence of an MM repair reduced the odds of achieving a PASS on the Pain (OR 0.59; 95% CI 0.36–0.96; *P* = 0.03) subscale. Hamstring tendon (HT) autograft rather than bone-patellar tendon-bone (BPTB) autograft showed increased odds (OR 2.02; 95% CI 1.31–3.10; *P* = 0.001), whereas a cartilage injury showed reduced odds (OR 0.73; 95% CI 0.55–0.97; *P* = 0.03) of achieving a PASS on the sport and recreation subscale. An LSI of ≥ 90% for 6-month single-leg-hop test performance increased the odds of achieving a PASS on the ADL (OR 1.37; 95% CI 1.09–1.71; *P* = 0.005), Sport and Recreation (OR 1.40; 95% CI 1.11–1.77; *P* = 0.004), and quality of life (OR 1.28; 95% CI 1.00–1.63; *P* = 0.04) subscales.

**Conclusion:**

More than 60% of the patients reported an acceptable symptom state on four of the five KOOS subscales 2 years after primary ACLR. Age ≥ 30 years and female gender were the non-modifiable factors that consistently increased and reduced, respectively, the odds of achieving a PASS. A symmetrical 6-month isokinetic quadriceps strength and single-leg-hop test performance were the modifiable factors that consistently increased the opportunity of achieving a PASS 2 years after primary ACLR.

**Level of evidence:**

III.

## Introduction

Patient-reported outcomes (PROs) are essential in clinical research, as they measure the patient’s perception of treatment. The Knee injury and Osteoarthritis Outcome Score (KOOS) [34] is a PRO that is consistently reported in the literature to measure subjective knee function after anterior cruciate ligament (ACL) reconstruction (ACLR) [4, 8, 9, 16, 20, 37]. However, the interpretability of the KOOS is not straightforward. An absolute post-operative score that might be regarded as successful by the clinician may not correspond to a patient’s satisfactory knee function [27]. Recently, Muller et al. [27] established, for each KOOS subscale, the threshold values for the achievement of a patient-acceptable symptom state (PASS) from 1 to 5 years after primary ACLR. These values were defined by answering the question: “Taking into account all the activity you have during your daily life, your level of pain, and also your activity limitations and participation restrictions, do you consider the current state of your knee satisfactory?”. The establishment of post-operative KOOS values corresponding to “feeling well” from the patient’s own perception of treatment and identified with the achievement of a PASS may facilitate the interpretation of the KOOS after ACLR.

A variety of factors may affect the achievement of a PASS after ACLR and current knowledge regarding these factors is limited. The heterogeneity of patients’ pre-operative, intra-operative and post-operative variables makes it difficult to predict which patients will be able to achieve a PASS after primary ACLR. However, knowledge of the factors affecting the achievement of a PASS after ACLR is important. First, this information would be very valuable to counsel and advise patients about their future expectations. Moreover, if any of these factors is modifiable, this gives us the opportunity to make changes to our treatment plan with an anticipated improved outcome. A full understanding of the factors affecting the achievement of a PASS after primary ACLR, therefore, allows us to personalise and maximise our patient care. To date, no previous studies have assessed the rate of patients achieving a PASS after primary ACLR in a large cohort. In addition, a detailed analysis of the patient factors affecting its achievement has not previously been presented.

The purpose of this study was to assess the percentage of patients achieving a PASS 2 years after primary ACLR and, moreover, to identify pre-operative, intra-operative, and post-operative factors affecting its achievement, in a large cohort. The hypothesis was that age, gender, graft choice, concomitant meniscal surgery, or cartilage injuries and the recovery of 6-month symmetrical isokinetic quadriceps or hamstring strength and single-leg-hop test performance would affect the achievement of a PASS 2 years after primary ACLR.

## Materials and methods

A total of 5231 patients who underwent primary ACLR at Capio Artro Clinic, Stockholm, Sweden, from 2005 to 2015, with no concomitant ligament injuries, were identified. The exclusion criteria were contralateral ACL injuries or reconstruction (*n* = 227) and revision ACLR (*n* = 210) during the follow-up. A cohort of 4794 patients was thus eligible for inclusion in the study. From this cohort, 2459 patients (51.3%) were excluded due to no KOOS data at the 2-year follow-up.

### Surgical technique and rehabilitation

All the patients underwent surgery using a single-bundle autologous hamstring tendon (HT) or bone-patellar tendon-bone (BPTB) technique. For the ACLRs performed with HT graft, the semitendinosus tendon was primarily harvested and prepared as a triple or quadruple graft. If the length or the diameter of the graft was considered insufficient (< 8 mm), the gracilis tendon was additionally harvested and combined with the semitendinosus graft. The BPTB graft was harvested as the central third of the patellar tendon with two bone blocks. The femoral tunnel was drilled using an anteromedial portal technique. Both grafts were routinely fixed using an Endobutton fixation device (Smith & Nephew, Andover, Mass, USA) on the femoral side and Ethibond no. 2 sutures (Ethicon, Sommerville, NJ) tied over an AO bicortical screw with a washer as a post or using an interference screw on the tibial side. Meniscal repair was performed, for both the medial meniscus (MM) and lateral meniscus (LM), with an arthroscopic all-inside technique, using a Fast-Fix suture anchor device (Smith and Nephew, Andover, Mass, USA), or an inside-out technique for tears located in the dorsal and middle portion of the meniscus. An outside-in technique was used for tears located in the anterior portion of the meniscus. Both inside-out and outside-in meniscal repair techniques were performed using PDS 0 (Ethicon, Sommerville, NJ). All the patients followed a standardised post-operative rehabilitation protocol. In the event of an isolated ACLR or ACLR with simultaneous meniscal resection, full weight bearing and full range of motion were encouraged as tolerated. If meniscal repair was performed, patients wore a hinged knee brace for 6 weeks. Flexion was limited from 0° to 30° for the first 2 weeks, from 0° to 60° for the third and fourth weeks and from 0° to 90° for the fifth and sixth weeks after surgery. Starting from the seventh week, the knee brace was discontinued and progressive weight bearing was allowed. The early rehabilitation phase focused on regaining range of motion, reducing swelling, and correcting gait. The rehabilitation protocol included joint and muscle flexibility exercises, balance/coordination training, and strength training, focusing primarily on the thigh muscles. For all patients, quadriceps strengthening was restricted to closed kinetic chain exercises during the first 3 months. On the basis of muscle strength, coordination, hop performance, and sport practised, the patients were allowed to return to sports 6 months post-operatively at the earliest.

### Isokinetic strength and single-leg-hop test performance assessment

The patients underwent isokinetic strength and single-leg-hop test performance assessment using a standardised protocol 6 months post-operatively.

Isokinetic concentric quadriceps and hamstring strength were measured bilaterally at 90°/s using the Biodex System 3 (Biodex Medical Systems, Shirley, New York, USA). The test was performed in a range of motion between 90° and 10° of knee flexion, always starting with the contralateral uninjured knee. Prior to the test, the patients warmed up using a stationary cycling ergometer at low resistance for 10 min. Patients were given a verbal description of the test and two-to-three practical trials were allowed before testing. Each patient performed five maximum quadriceps and hamstring contractions with each leg. Patients were verbally encouraged during the test. The peak quadriceps and hamstring torque values (highest achieved values) were registered.

The single-leg-hop test was used to assess functional hop performance [31, 35]. The test was performed with the patient standing on one leg and being instructed to jump straight ahead as far as possible and land on the same leg. The test was considered successful if the landing was stable. If the patient landed with an early touchdown of the contralateral limb, which had loss of balance or took additional hops after landing, the hop was repeated. Patients were initially given a verbal description of the test and they were allowed to perform as many practical trials as they wanted, until they felt confident about the test. Three trials were performed for each leg, always starting with the contralateral uninjured leg. Patients were given as much time as they wanted between the trials to minimise fatigue. The best trial for each leg was registered.

The achievement of a symmetrical [limb symmetry index (LSI)] isokinetic quadriceps and hamstring strength or single-leg-hop test performance was defined as performing at least 90% of the uninvolved limb (LSI ≥ 90%) for each test [13, 29, 44].

### Data sources

Demographic data (age and gender), information about the time from injury to surgery, pre-injury Tegner activity level [43], graft type, meniscus surgery, the presence of cartilage injuries, and the results of the isokinetic quadriceps and hamstring strength tests and single-leg-hop test 6 months after ACLR were collected in our clinic registry. Meniscus surgery was classified as follows: no meniscus surgery, meniscus resection, or meniscus repair for both the medial and lateral meniscus. The results of the KOOS at the 2-year follow-up were reviewed.

### Outcome

The primary outcome of the study was the achievement of a PASS for each KOOS subscale [27] 2 years after ACLR. The KOOS is a frequently used disease-specific PRO for measuring functional knee outcome in patients undergoing ACLR. It is divided into five subscales: Pain, Knee-related Symptoms, Activities of Daily Living (ADL), Sport and Recreation, and Knee-related Quality of Life (QoL). The Sport and Recreation and QoL subscales have been reported by Roos et al. [34] to be the most responsive at a post-operative follow-up after ACLR.

Each subscale is scored from 0, representing “extreme knee problems”, to 100, representing “no knee problems”. It is recommended to evaluate the individual subscales independently [34]. The achievement of a PASS on the KOOS was assessed on the basis of the threshold values identified by Muller et al. [27]. The corresponding PASS values for the KOOS subscales were as follows: pain ≥ 88.9; symptoms ≥ 57.1; ADL = 100; sport and recreation ≥ 75.0; and QoL ≥ 62.5.

Ethical approval for this study was obtained from the regional ethics committee, Karolinska Institutet (Diarienumber 2016/1613-31/32).

### Statistical analysis

The Statistical Package for Social Sciences, SPSS (Version 25.0, IBM Corp., Armonk, New York, USA), was used for the statistics. All the variables were summarised with the standard descriptive statistics such as the mean, standard deviations (SD), or frequency. The distributions were checked for severe skewness (> 1.5) and outliers. To compare the included (with 2-year KOOS data) and the excluded (with no 2-year KOOS data) patients (dropout analysis), Pearson’s Chi-square test was used.

The achievement of a PASS for each KOOS subscale was used separately as outcome measurement. Multivariate logistic regression analyses were performed with age, gender (female vs. male), time from injury to surgery (delayed > 3 months vs. not delayed ≤ 3 months), pre-injury Tegner activity level (high ≥ 6 vs. low < 6), graft (HT vs. BPTB autograft), medial meniscus resection, medial meniscus repair, lateral meniscus resection, lateral meniscus repair, cartilage injury and the recovery of 6-month symmetrical (LSI ≥ 90%) isokinetic quadriceps strength, hamstring strength, and single-leg-hop test performance as independent variables, and the achievement of a PASS on each KOOS subscale as the dependent variable. Age was dichotomized into classes close to the median (≥ 30 years vs. < 30 years). The results of the logistic regression analyses were expressed as odds ratios (OR) with 95% confidence intervals (CI). The level of significance in all analyses was 5% (two-tailed).

Provided that there was a significance level of 5%, a power of 85%, and a sample size of more than 1000 patients, even a very weak relationship of less than 0.10 (phi coefficient), which corresponds to an effect size of less than 0.10, according to Cohen, would be detected.

## Results

A total of 2335 patients fulfilled the inclusion criteria with a complete 2-year post-operative follow-up for all the KOOS subscales. The percentage of 2-year KOOS outcome follow-up was 48.7% (2335/4794). A comparison between the included cohort and the cohort with no 2-year KOOS data (dropout analysis) is detailed in Table 1. Patients with no 2-year KOOS data were significantly younger (*P *< 0.001). Although the difference in mean age between the groups was only 1.4 years, the patients in the included cohort were more likely to be 30 years old or older in comparison to patients with no 2-year KOOS data (47.2% vs. 40.1%). In addition, men were significantly more likely than women to be lost to follow-up (< 0.001). Female patients were significantly more represented in the included cohort in comparison to the cohort with no 2-year KOOS data (49.4% vs. 38.7%). The number of patients with a concomitant lateral meniscus resection was slightly lower in the included cohort in comparison to the excluded cohort (13.9% vs. 16.1%; *P *< 0.04). Meanwhile, no significant differences were found between the cohorts with regard to all the other variables (Table 1).

**Table 1:** Patient characteristics and dropout analysis

	Included cohort (*n* = 2335)	No 2-year KOOS data (*n* = 2459)	*P* value
*Pre*-*operative factors*			
Age at surgery, years, mean ± SD	29.7 ± 10.9	28.3 ± 9.9	< 0.001
Age younger than 30 years	20.9 ± 4.6; 1233 (52.8)	21.4 ± 4.6; 1475 (59.9)	
Age 30 years or older	39.5 ± 6.7; 1102 (47.2)	38.5 ± 6.4; 984 (40.1)	
Gender			< 0.001
Male	1182 (50.6)	1508 (61.3)	
Female	1153 (49.4)	951 (38.7)	
Time from injury to surgery, months, mean ± SD	15.1 ± 8.7	16.9 ± 9.8	n.s.
≤ 3 months	381 (17.7)	352 (16.2)	
> 3 months	1769 (82.3)	1821 (83.8)	
	*n* = 2150	*n* = 2173	
Pre-injury Tegner activity level, median (range)	7 (1–10)	7 (1–10)	n.s.
High, ≥ 6	1749 (87.4)	1897 (89.1)	
Low, < 6	252 (12.6)	232 (10.9)	
	*n* = 2001	*n* = 2129	
*Intra*-*operative factors*			
Graft type			n.s.
HT autograft	2100 (89.9)	2182 (88.7)	
BPTB autograft	235 (10.1)	277 (11.3)	
No meniscus surgery	1528 (65.4)	1473 (59.9)	
Medial meniscus surgery			
Resection	321 (13.7)	379 (15.4)	n.s.
Repair	95 (4.0)	128 (5.2)	n.s.
Lateral meniscus surgery			
Resection	326 (13.9)	395 (16.1)	0.04
Repair	65 (2.8)	84 (3.4)	n.s.
Cartilage injury			n.s.
Yes	469 (20.0)	478 (19.4)	
No	1866 (80.0)	1981 (80.6)	
*Post*-*operative factors* (*6* *months*)			
Isokinetic quadriceps strength			n.s.
LSI ≥ 90%	758 (32.5)	713 (34.3)	
LSI < 90%	1575 (67.5)	1367 (65.7)	
	*n* = 2333	*n* = 2080	
Isokinetic hamstring strength			n.s.
LSI ≥ 90%	1120 (48.0)	963 (46.3)	
LSI < 90%	1210 (52.0)	1115 (53.7)	
	*n* = 2330	*n* = 2078	
Single-leg-hop test			n.s.
LSI ≥ 90%	1335 (65.5)	1210 (66.9)	
LSI < 90%	704 (34.5)	596 (33.1)	
	*n* = 2039	*n* = 1806	

Data are reported as *n* (%), unless otherwise indicated

*KOOS* Knee injury and Osteoarthritis Outcome Score; *SD* standard deviation; *HT* hamstring tendon; *BPTB* bone-patellar tendon bone; *LSI* limb symmetry index

The 2-year mean KOOS values and the rate of PASS for each dichotomized patient group in the included cohort are detailed in Table 2.

**Table 2:** Mean ± SD (PASS %) KOOS values for each dichotomized patient group in the included cohort (*n* = 2335 patients) at the 2-year follow-up

	Pain (PASS ≥ 88.9)	Symptoms (PASS ≥ 57.1)	ADL (PASS = 100)	Sport and recreation (PASS ≥ 75.0)	QoL (PASS ≥ 62.5)
*Pre*-*operative factors*					
Age at surgery					
Age younger than 30 years	87.9 ± 13.9 (56.4)	81.4 ± 16.5 (92.3)	94.2 ± 10.9 (44.6)	73.4 ± 24.5 (61.3)	65.6 ± 23.8 (64.5)
Age 30 years or older	90.3 ± 12.3 (64.0)	85.4 ± 14.4 (95.2)	94.5 ± 10.4 (46.7)	74.9 ± 24.7 (64.3)	71.0 ± 21.4 (74.1)
Gender					
Male	89.9 ± 13.2 (71.5)	83.6 ± 15.9 (93.8)	94.5 ± 10.9 (49.0)	76.3 ± 23.5 (66.3)	69.4 ± 23.3 (70.6)
Female	88.1 ± 13.2 (65.0)	82.9 ± 15.4 (93.6)	94.2 ± 10.4 (42.1)	71.8 ± 25.5 (59.0)	66.8 ± 22.4 (67.4)
Time from injury to surgery					
≤ 3 months	89.0 ± 13.1 (69.1)	82.3 ± 16.5 (92.8)	94.6 ± 10.6 (48.8)	74.8 ± 24.5 (64.2)	68.1 ± 23.2 (69.7)
> 3 months	89.0 ± 13.2 (67.9)	83.6 ± 15.4 (94.0)	94.3 ± 10.7 (44.5)	73.9 ± 24.7 (62.2)	68.2 ± 22.8 (68.8)
Pre-injury Tegner activity level					
High ≥ 6	89.0 ± 13.0 (67.8)	83.0 ± 15.7 (93.4)	94.5 ± 10.3 (45.4)	74.6 ± 24.0 (62.9)	68.1 ± 22.7 (68.9)
Low < 6	89.0 ± 13.7 (69.6)	83.9 ± 15.0 (94.2)	94.0 ± 11.6 (46.2)	72.8 ± 26.3 (61.2)	68.3 ± 23.4 (69.4)
*Intra*-*operative factors*					
Graft type					
HT autograft	89.2 ± 12.8 (68.5)	83.4 ± 15.0 (93.7)	94.5 ± 10.3 (45.1)	74.7 ± 24.2 (63.4)	68.5 ± 22.5 (69.4)
BPTB autograft	87.5 ± 16.0 (65.9)	82.1 ± 18.1 (92.3)	93.1 ± 13.6 (49.8)	69.0 ± 28.0 (56.1)	65.0 ± 25.8 (65.5)
No meniscus surgery	89.1 ± 13.0 (68.6)	83.6 ± 15.6 (93.8)	94.4 ± 10.5 (45.7)	74.2 ± 24.6 (62.5)	68.5 ± 23.0 (70.3)
Medial meniscus surgery					
Resection	89.9 ± 14.0 (73.2)	85.0 ± 13.8 (96.2)	94.6 ± 11.5 (49.2)	75.8 ± 23.8 (66.7)	69.8 ± 21.7 (71.0)
Repair	85.3 ± 14.0 (54.7)	78.0 ± 16.5 (88.4)	93.4 ± 10.9 (37.8)	68.0 ± 27.6 (55.8)	62.3 ± 23.4 (57.9)
Lateral meniscus surgery					
Resection	89.0 ± 14.0 (66.6)	82.4 ± 16.2 (92.3)	93.9 ± 12.5 (43.9)	74.3 ± 24.1 (63.8)	67.9 ± 23.0 (67.8)
Repair	87.9 ± 13.0 (66.2)	79.2 ± 20.0 (87.6)	96.3 ± 6.1 (50.7)	73.7 ± 26.5 (64.6)	67.5 ± 23.1 (64.6)
Cartilage injury					
Yes	89.2 ± 12.0 (68.0)	82.9 ± 15.3 (93.6)	94.1 ± 10.2 (42.2)	72.3 ± 25.7 (59.3)	68.1 ± 22.4 (69.1)
No	89.0 ± 13.0 (68.4)	83.4 ± 15.8 (93.7)	94.4 ± 10.8 (46.1)	74.5 ± 24.3 (63.6)	68.2 ± 23.0 (69.1)
*Post*-*operative factors* (*6* *months*)					
Isokinetic quadriceps strength					
LSI ≥ 90%	90.3 ± 12.6 (73.6)	85.3 ± 15.0 (95.4)	95.6 ± 9.5 (51.7)	78.1 ± 23.1 (70.5)	71.2 ± 22.5 (74.5)
LSI < 90%	88.4 ± 13.0 (65.8)	82.3 ± 16.0 (92.8)	93.8 ± 11.1 (42.6)	72.1 ± 25.1 (58.9)	66.7 ± 22.9 (66.4)
Isokinetic hamstring strength					
LSI ≥ 90%	89.4 ± 13.0 (70.4)	83.4 ± 15.9 (93.2)	94.7 ± 10.9 (47.7)	75.5 ± 24.3 (65.2)	69.0 ± 23.0 (70.7)
LSI < 90%	88.7 ± 13.0 (66.3)	83.2 ± 15.5 (93.9)	94.0 ± 10.4 (43.6)	72.8 ± 24.8 (60.3)	67.4 ± 22.8 (67.5)
Single-leg-hop test					
LSI ≥ 90%	90.0 ± 12.0 (70.6)	84.1 ± 15.5 (94.4)	95.5 ± 8.9 (49.5)	77.5 ± 22.4 (67.7)	70.2 ± 22.3 (72.1)
LSI < 90%	87.7 ± 14.0 (65.1)	82.1 ± 15.9 (92.6)	92.9 ± 12.5 (40.2)	69.6 ± 26.6 (55.9)	65.3 ± 23.2 (64.9)

*KOOS* Knee injury and Osteoarthritis Outcome Score; *PASS* patient-acceptable symptom state; *ADL* activities of daily living; *QoL* quality of life; *SD* standard deviation; *HT* hamstring tendon; *BPTB* bone-patellar tendon bone; *LSI* limb symmetry index

The proportion of patients achieving a PASS varied between the KOOS subscales as follows: pain 68.3%; symptoms 93.6%; ADL 45.6%; sport and recreation 62.6%; and QoL 69.0%.

Age ≥ 30 years and an LSI of ≥ 90% for 6-month isokinetic quadriceps strength increased the odds of achieving a PASS across all KOOS subscales. Female gender reduced the odds of achieving a PASS on the Pain, ADL, and Sport and Recreation subscales. The presence of an MM repair reduced the odds of achieving a PASS on the Pain subscale. The use of HT autograft rather than BPTB autograft showed increased odds, whereas a cartilage injury showed reduced odds of achieving a PASS on the Sport and Recreation subscale. An LSI of ≥ 90% for 6-month single-leg-hop test performance increased the odds of achieving a PASS on the ADL, Sport and Recreation, and QoL subscales. No other factors were found to be associated with the achievement of a PASS on the KOOS subscales 2 years after ACLR (Tables 3, 4, 5, 6, and 7).

**Table 3:** Factors affecting the achievement of a PASS on the KOOS pain (≥ 88.9) subscale

Factor	Regression coefficient (ß)	SE	OR (95% CI)	*P* value
*Pre*-*operative*				
Age ≥ 30 years	0.43	0.11	1.54 (1.23–1.92)	< 0.001*
Female gender	− 0.26	0.10	0.76 (0.62–0.94)	0.01*
Delayed (> 3 months) ACLR	0.04	0.13	1.04 (0.80–1.36)	n.s.
Pre-injury Tegner activity level ≥ 6	− 0.01	0.16	0.98 (0.71–1.36)	n.s.
*Intra*-*operative*				
HT autograft	0.29	0.21	1.34 (0.88–2.05)	n.s.
MM resection	0.18	0.15	1.19 (0.88–1.62)	n.s.
MM repair	− 0.52	0.24	0.59 (0.36–0.96)	0.03*
LM resection	− 0.11	0.14	0.88 (0.66–1.18)	n.s.
LM repair	0.21	0.30	1.23 (0.68–2.24)	n.s.
Cartilage injury	0.05	0.14	1.00 (0.76–1.32)	n.s.
*Post*-*operative* (*6* *months*)				
Quadriceps strength LSI ≥ 90%	0.30	0.11	1.35 (1.08–1.70)	0.009*
Hamstring strength LSI ≥ 90%	− 0.02	0.10	0.97 (0.79–1.20)	n.s.
Single-leg-hop test LSI ≥ 90%	0.14	0.11	1.15 (0.92–1.44)	n.s.

*PASS* Patient-acceptable symptom state; *KOOS* Knee injury and Osteoarthritis Outcome Score; *ACLR* anterior cruciate ligament reconstruction; *HT* hamstring tendon; *MM* medial meniscus; *LM* lateral meniscus; *LSI* limb symmetry index; *SE* standard error; *OR* odds ratio; *CI* confidence interval

*Statistically significant. *P* value < 0.05

**Table 4:** Factors affecting the achievement of a PASS on the KOOS Symptoms (≥ 57.1) subscale

Factor	Regression coefficient (ß)	SE	OR (95% CI)	*P* value
*Pre*-*operative*				
Age ≥ 30 years	0.52	0.23	1.68 (1.06–2.67)	0.02*
Female gender	− 0.07	0.20	0.92 (0.61–1.39)	n.s.
Delayed (> 3 months) ACLR	0.23	0.24	1.26 (0.78–2.05)	n.s.
Pre-injury Tegner activity level ≥ 6	− 0.24	0.36	0.78 (0.37–1.61)	n.s.
*Intra*-*operative*				
HT autograft	0.30	0.36	1.35 (0.66–2.78)	n.s.
MM resection	0.72	0.40	2.06 (0.93–4.56)	n.s.
MM repair	− 0.22	0.42	0.79 (0.34–1.84)	n.s.
LM resection	− 0.39	0.27	0.67 (0.39–1.14)	n.s.
LM repair	− 0.49	0.46	0.60 (0.24–1.51)	n.s.
Cartilage injury	0.16	0.29	1.81 (0.65–2.12)	n.s.
*Post*-*operative* (*6 months*)				
Quadriceps strength LSI ≥ 90%	0.48	0.24	1.62 (1.00–2.63)	0.04*
Hamstring strength LSI ≥ 90%	− 0.19	0.21	0.82 (0.54–1.24)	n.s.
Single-leg-hop test LSI ≥ 90%	0.34	0.22	1.41 (0.91–2.17)	n.s.

*PASS* Patient-acceptable symptom state; *KOOS* Knee injury and Osteoarthritis Outcome Score; *ACLR* anterior cruciate ligament reconstruction; *HT* hamstring tendon; *MM* medial meniscus; *LM* lateral meniscus; *LSI* limb symmetry index; *SE* standard error; *OR* odds ratio; *CI* confidence interval

*Statistically significant. *P* value < 0.05

**Table 5:** Factors affecting the achievement of a PASS on the KOOS ADL (= 100.0) subscale

Factor	Regression coefficient (ß)	SE	OR (95% CI)	*P* value
*Pre*-*operative*				
Age ≥ 30 years	0.32	0.11	1.37 (1.10–1.71)	0.004*
Female gender	− 0.23	0.10	0.79 (0.64–0.97)	0.02*
Delayed (> 3 months) ACLR	0.20	0.13	0.81 (0.62–1.06)	n.s.
Pre-injury Tegner activity level ≥ 6	0.02	0.16	1.00 (0.72–1.38)	n.s.
*Intra*-*operative*				
HT autograft	0.01	0.21	1.01 (0.66–1.55)	n.s.
MM resection	0.19	0.14	1.21 (0.90–1.61)	n.s.
MM repair	− 0.20	0.25	0.81 (0.49–1.33)	n.s.
LM resection	− 0.20	0.14	0.81 (0.61–1.08)	n.s.
LM repair	0.47	0.29	1.60 (0.89–2.86)	n.s.
Cartilage injury	− 0.12	0.13	0.88 (0.67–1.15)	n.s.
*Post*-*operative* (*6* *months*)				
Quadriceps strength LSI ≥ 90%	0.29	0.11	1.34 (1.07–1.67)	0.01*
Hamstring strength LSI ≥ 90%	− 0.03	0.10	0.97 (0.79–1.19)	n.s.
Single-leg-hop test LSI ≥ 90%	0.31	0.11	1.37 (1.09–1.71)	0.005*

*PASS* Patient-acceptable symptom state; *KOOS* Knee injury and Osteoarthritis Outcome Score; *ADL* activities of daily living; *ACLR* anterior cruciate ligament reconstruction; *HT* hamstring tendon; *MM* medial meniscus; *LM* lateral meniscus; *LSI* limb symmetry index; *SE* standard error; *OR* odds ratio; *CI* confidence interval

*Statistically significant. *P* value < 0.05

**Table 6:** Factors affecting the achievement of a PASS on the KOOS Sport and Recreation (≥ 75.0) subscale

Factor	Regression coefficient (ß)	SE	OR (95% CI)	*P* value
*Pre*-*operative*				
Age ≥ 30 years	0.41	0.11	1.52 (1.20–1.91)	< 0.001*
Female gender	− 0.32	0.11	0.72 (0.58–0.89)	0.003*
Delayed (> 3 months) ACLR	− 0.15	0.14	0.86 (0.64–1.14)	n.s.
Pre-injury Tegner activity level ≥ 6	− 0.30	0.17	0.73 (0.52–1.04)	n.s.
*Intra*-*operative*				
HT autograft	0.70	0.21	2.02 (1.31–3.10)	0.001*
MM resection	0.13	0.16	1.14 (0.83–1.57)	n.s.
MM repair	− 0.18	0.25	0.82 (0.50–1.36)	n.s.
LM resection	− 0.04	0.15	0.96 (0.70–1.30)	n.s.
LM repair	0.36	0.32	1.44 (0.76–2.70)	n.s.
Cartilage injury	− 0.30	0.14	0.73 (0.55–0.97)	0.03*
*Post*-*operative* (*6* *months*)				
Quadriceps strength LSI ≥ 90%	0.41	0.12	1.51 (1.18–1.92)	0.001*
Hamstring strength LSI ≥ 90%	0.06	0.11	1.06 (0.85–1.32)	n.s.
Single-leg-hop test LSI ≥ 90%	0.34	0.11	1.40 (1.11–1.77)	0.004*

*PASS* Patient-acceptable symptom state; *KOOS* Knee injury and Osteoarthritis Outcome Score; *ACLR* anterior cruciate ligament reconstruction; *HT* hamstring tendon; *MM* medial meniscus; *LM* lateral meniscus; *LSI* limb symmetry index; *SE* standard error; *OR* odds ratio; *CI* confidence interval

*Statistically significant. *P* value < 0.05

**Table 7:** Factors affecting the achievement of a PASS on the KOOS QoL (≥ 62.5) subscale

Factor	Regression coefficient (ß)	SE	OR (95% CI)	*P* value
*Pre*-*operative*				
Age ≥ 30 years	0.82	0.12	2.28 (1.78–2.92)	< 0.001*
Female gender	− 0.13	0.11	0.87 (0.69–1.09)	n.s.
Delayed (> 3 months) ACLR	− 0.09	0.14	0.90 (0.67–1.21)	n.s.
Pre-injury Tegner activity level ≥ 6	− 0.08	0.18	0.91 (0.63–1.31)	n.s.
*Intra*-*operative*				
HT autograft	0.25	0.22	1.29 (0.82–2.01)	n.s.
MM resection	− 0.09	0.16	0.99 (0.71–1.38)	n.s.
MM repair	− 0.38	0.25	0.68 (0.41–1.12)	n.s.
LM resection	0.06	0.16	1.06 (0.77–1.47)	n.s.
LM repair	0.25	0.32	1.28 (0.67–2.43)	n.s.
Cartilage injury	− 0.01	0.15	0.98 (0.72–1.32)	n.s.
*Post*-*operative* (*6* *months*)				
Quadriceps strength LSI ≥ 90%	0.39	0.12	1.48 (1.15–1.91)	0.002*
Hamstring strength LSI ≥ 90%	0.02	0.11	1.02 (0.81–1.28)	n.s.
Single-leg-hop test LSI ≥ 90%	0.24	0.12	1.28 (1.00–1.63)	0.04*

*PASS* Patient-acceptable symptom state; *KOOS* Knee injury and Osteoarthritis Outcome Score; *QoL* quality of life; *ACLR* anterior cruciate ligament reconstruction; *HT* hamstring tendon; *MM* medial meniscus; *LM* lateral meniscus; *LSI* limb symmetry index; *SE* standard error; *OR* odds ratio; *CI* confidence interval

*Statistically significant. *P* value < 0.05

## Discussion

The main finding of this study was that the proportion of patients achieving a PASS (i.e., “feeling well”) 2 years after ACLR varied substantially between the KOOS subscales, from 45.6% for the ADL subscale to 93.6% for the Symptoms subscale. However, more than 60% of the patients reported a PASS on four of the five KOOS subscales. The largest effect on predicting the achievement of a PASS on the different KOOS subscales 2 years after ACLR was found for age, gender, and the recovery of 6-month symmetrical isokinetic quadriceps strength and single-leg-hop test performance. Older age (≥ 30 years) consistently increased the odds of achieving a PASS across all KOOS subscales, whereas female gender reduced the odds of achieving a PASS on the Pain, ADL, and Sport and Recreation subscales. The recovery of 6-month symmetrical isokinetic quadriceps strength increased the odds of achieving a PASS on all KOOS subscales and a symmetrical 6-month single-leg-hop test performance increased the odds of achieving a PASS on the ADL, Sport and Recreation, and QoL subscales 2 years after ACLR.

The previous studies have investigated the effect of age on subjective knee function, measured with the KOOS, after primary ACLR [1, 9, 12]. However, they reported conflicting results. Ageberg et al. [1] found that, at 2 years post-operatively, age did not influence the KOOS scores. Hamrin Senorski et al. [12], in a recent study based on 343 patients, found that younger age at reconstruction results in favourable odds of achieving a PASS across the KOOS subscales 1 year after ACLR. On the other hand, Desai et al. [9], in a previous, larger study based on the Swedish national knee ligament registry, showed that older age is associated with better subjective knee function, measured with the KOOS, after ACLR. Our study confirms these findings, showing that age ≥ 30 years consistently increases the odds of achieving a PASS across all the KOOS subscales. Younger patients (< 30 years) could constitute a more active population, more likely to expose their knees to loads and activities requiring high function. As a result, they might not be completely satisfied more frequently after surgery, reporting a lower KOOS [9] and reducing the odds of achieving a PASS post-operatively.

Several studies have reported inferior outcomes for females after ACLR [1, 9, 12, 42]. Ageberg et al. [1] showed that female patients report poorer scores than male patients on the KOOS Sport and Recreation and KOOS QoL, at 2 years post-operatively. They hypothesised that one possible reason for female patients reporting poorer outcomes than male patients might be differences in muscle function. However, the effect of muscle function, expressed as symmetrical (LSI ≥ 90%) 6-month isokinetic quadriceps and hamstring strength and single-leg-hop test performance, was taken into account in our study. These three factors were included as separate dependent variables in our multivariate logistic regression analysis. Nevertheless, our results showed that female gender is a factor that is per se negatively associated with the achievement of a PASS on the KOOS Pain, ADL, and Sport and Recreation subscales 2 years after primary ACLR. It is not clear why female patients report poorer subjective knee outcomes. A recent systematic review and meta-analysis [42], including a total of 135 publications with more than 120.000 patients, revealed that females report poorer subjective outcomes than males after ACLR. However, no difference was found in any objective parameter.

One unanticipated result was that a longer (> 3 months) time interval from injury to surgery per se had no effect on the achievement of a PASS on any of the KOOS subscale. Several studies have suggested potential benefits from attempting to shorten the time span between injury and ACLR [19, 45]. A longer waiting time from injury to surgery may increase the risk of additional cartilage and medial meniscus injuries in the ACL-deficient knee [3]. However, it is possible that delayed surgery per se does not impact subjective knee outcome after ACLR, unless recurrent giving ways with subsequent meniscal or cartilage injuries occur.

The use of HT autograft rather than BPTB autograft was associated with increased odds of achieving a PASS on the KOOS Sport and Recreation subscale. This difference between the grafts could be explained by the “donor-site morbidity” associated with the BPTB autograft [5, 8, 10, 24, 46]. In a large cohort study based on the Swedish national knee ligament registry, Barenius et al. [3] showed that the use of BPTB autograft is a negative predictor of functional recovery 2 years after ACLR.

Interestingly, neither MM nor LM resection affected the achievement of a PASS on any of the KOOS subscales 2 years after primary ACLR. However, it is known that a concurrent meniscal resection may negatively affect the long-term outcomes after ACLR [4, 6, 28, 38, 40]. Similarly, in the present study, the presence of a cartilage injury at the time of primary ACLR only weakly affected (*P *= 0.03) the achievement of a PASS on the Sport and Recreation KOOS subscale at the 2-year follow-up. No correlation between the presence of a cartilage injury and the achievement of a PASS on any of the other KOOS subscales was found. Nevertheless, the association between cartilage injury and an inferior KOOS on several subscales at a long-term follow-up has been previously established [7, 14, 33]. Perhaps, a 2-year follow-up is too early to see the reported poorer long-term outcomes related to meniscal resection or the presence of a cartilage injury. The consequences of losing meniscal tissue on subjective knee function may not be appreciated until repetitive loading on the knee occurs over the course of several years after ACLR [22]. The same consideration could be applied to cartilage injuries, with symptoms that may not be revealed in the first few years after ACLR.

The presence of a concomitant MM repair reduced the odds of achieving a PASS on the KOOS Pain subscale. Few recent studies [22, 30, 41] have attempted to clarify the effects of concomitant meniscal resection or repair on post-operative outcomes after ACLR at a short-term follow-up. Svantesson et al. [41] reported that patients with meniscal repair have poorer subjective knee function, measured with the Lysholm and KOOS, at both the 6- and the 12-month follow-up after ACLR. LaPrade et al. [22] reported that the 2-year post-operative KOOS in patients with MM resection, LM resection or LM repair did not differ significantly from an isolated ACLR for any of the five KOOS subscales. On the other hand, the results after an MM repair were significantly inferior for the Symptoms and QoL KOOS subscales. It has been suggested [22] that the decreased mobility of the MM in comparison with the LM and the different insertion geometries of the medial and lateral meniscus roots [18, 23, 45] may explain the better outcomes for LM repair in comparison with MM repair after ACLR at a short-term follow-up.

The recovery of symmetrical isokinetic quadriceps and hamstring strength and single-leg-hop test performance is regarded as a key factor prior to return to sport after ACLR. Muscular and functional asymmetries are known to be risk factors for ACL graft tears and knee re-injuries [11, 15, 21]. However, only a few studies [17, 25, 39] have attempted to analyse the effects of muscular strength and hop performance on longitudinal subjective knee outcome after ACLR. In the present study, the recovery of 6-month symmetrical isokinetic quadriceps strength consistently increased the odds of achieving a PASS on all the KOOS subscales and a symmetrical 6-month single-leg-hop test performance increased the odds of achieving a PASS on the ADL, Sport and Recreation, and QoL subscales 2 years after ACLR. Among all the modifiable factors studied, the results of these tests had the greatest effect on predicting the achievement of a PASS on the different KOOS subscales 2 years after ACLR. Conducting these tests 6 months after ACLR could, therefore, be appropriate for predictive purposes, as they can be used to inform the clinician about the patient’s likely prognosis and the need for targeted rehabilitation to address strength and hop asymmetries and improve subjective knee function, promoting the achievement of a PASS after ACLR.

The results of the present study have significant implications for the clinical management of patients after primary ACLR. This is, to our knowledge, the first large cohort study assessing the percentage of patients achieving an acceptable symptom state 2 years after primary ACLR. In addition, it provides unique data, comprising a detailed analysis of patient pre-operative, intra-operative and post-operative factors affecting the achievement of a PASS 2 years after primary ACLR. To improve treatment outcome after ACLR and assess individual expected outcome, a large spectrum of potential factors affecting subjective knee outcome must be simultaneously evaluated [26]. Some key factors, like age and gender, are non-modifiable. However, an awareness of the effect of these factors on the achievement of a PASS could help clinicians to counsel patients about their expectations after ACLR. On the other hand, most of the surgery- and rehabilitation-related factors are modifiable. The knowledge of these modifiable factors should, therefore, be used by clinicians and physical therapists to improve subjective knee function and maximise the achievement of a PASS after primary ACLR.

The main strength of this study is the analysis of a large cohort (2335 patients). This enabled a robust logistic regression analysis and a detailed, comprehensive evaluation of several factors affecting the achievement of a PASS after primary ACLR that have not been investigated in the previous studies. The study cohort represented a wide range of patients in terms of age, time from injury to surgery, pre-injury activity level, and concomitant meniscal surgery. The results of this study are, therefore, highly generalizable. All the patients received standardised surgery, rehabilitation, and post-operative assessment at the same institution. Finally, this study analysed the results of the KOOS after ACLR in a clinically meaningful way. The achievement of a PASS is known to correlate with the patient’s perception of treatment [27].

The main limitation is the suboptimal follow-up. Only 48.7% of the patients had filled in the KOOS questionnaires 2 years post-operatively. The loss of approximately 51% of patients due to missing 2-year KOOS values is not ideal, but this follow-up rate is in line with the previous large cohort studies [2, 20]. The patients lost to follow-up tended to be younger and with a larger proportion of men than women in the included cohort. This phenomenon has previously been described in a non-response analysis of 2-year data in the Swedish national knee ligament registry [32]. Differences between the patients included and lost to follow-up, in terms of age and gender, may have the potential for selection bias and could have influenced the results. However, with the exception of age and gender, all the other patient characteristics were comparable between the included cohort and the cohort lost to follow-up. The lack of details regarding the depth and location of cartilage injuries is a limitation. Røtterud et al. [36] found no significant associations between partial-thickness cartilage lesions and the scores on any of the KOOS subscales at the 2-year follow-up. On the other hand, full-thickness cartilage lesions were significantly associated with reduced scores on all the KOOS subscales. It is possible that the dichotomization of cartilage injury to “yes” or “no” in our study has acted as a confounder for predicting the achievement of a PASS after ACLR. The same consideration could be applied to meniscal resections. Unfortunately, information regarding the size and location of meniscal resections was not available. It could be hypothesised that larger resections have a greater impact on PROs at follow-up.

## Conclusion

More than 60% of the patients reported an acceptable symptom state on four of the five KOOS subscales 2 years after primary ACLR. Age ≥ 30 years and female gender were the non-modifiable factors that consistently increased and reduced, respectively, the odds of achieving a PASS. Symmetrical 6-month isokinetic quadriceps strength and single-leg-hop test performance were the modifiable factors that consistently increased the opportunity of achieving a PASS 2 years after primary ACLR.
